# Pathological Characteristics of the Vascular Septum in Chronic Aortic Dissection: A Case Report

**DOI:** 10.7759/cureus.48910

**Published:** 2023-11-16

**Authors:** Tomohiro Nakajima, Ayaka Arihara, Kei Mukawa, Yutaka Iba, Nobuyoshi Kawaharada

**Affiliations:** 1 Cardiovascular Surgery, Sapporo Medical University, Sapporo, JPN

**Keywords:** limb ischemia, type b acute aortic dissection, pathology, vascular septum, chronic dissection

## Abstract

A 46-year-old male developed a Stanford type B aortic dissection. At age 48, he underwent left open thoracic descending aorta replacement because of the enlargement of the descending thoracic aorta. At 51 years old, he underwent abdominal aorta replacement because of ischemia in the right lower extremity and the enlargement of an abdominal aortic aneurysm. The septum between the true and false lumens was submitted to histopathological examination, which revealed bilateral intimal tissue with the tunica media lying in between.

## Introduction

In some cases, acute aortic dissection is followed by chronic aortic dissection or aneurysm [1]. The wall separating the true and false lumens gradually thickens in this process. Few papers have described the pathogenesis of these wall changes. In this study, we discuss the underlying pathology in such cases.

## Case presentation

A 51-year-old male developed acute aortic type B dissection five years previously. At 48 years of age, he underwent descending thoracic aorta graft replacement with a 28-mm J-graft (Japan Lifeline Inc., Tokyo, Japan) for the management of aortic dilation.

**Figure 1:**
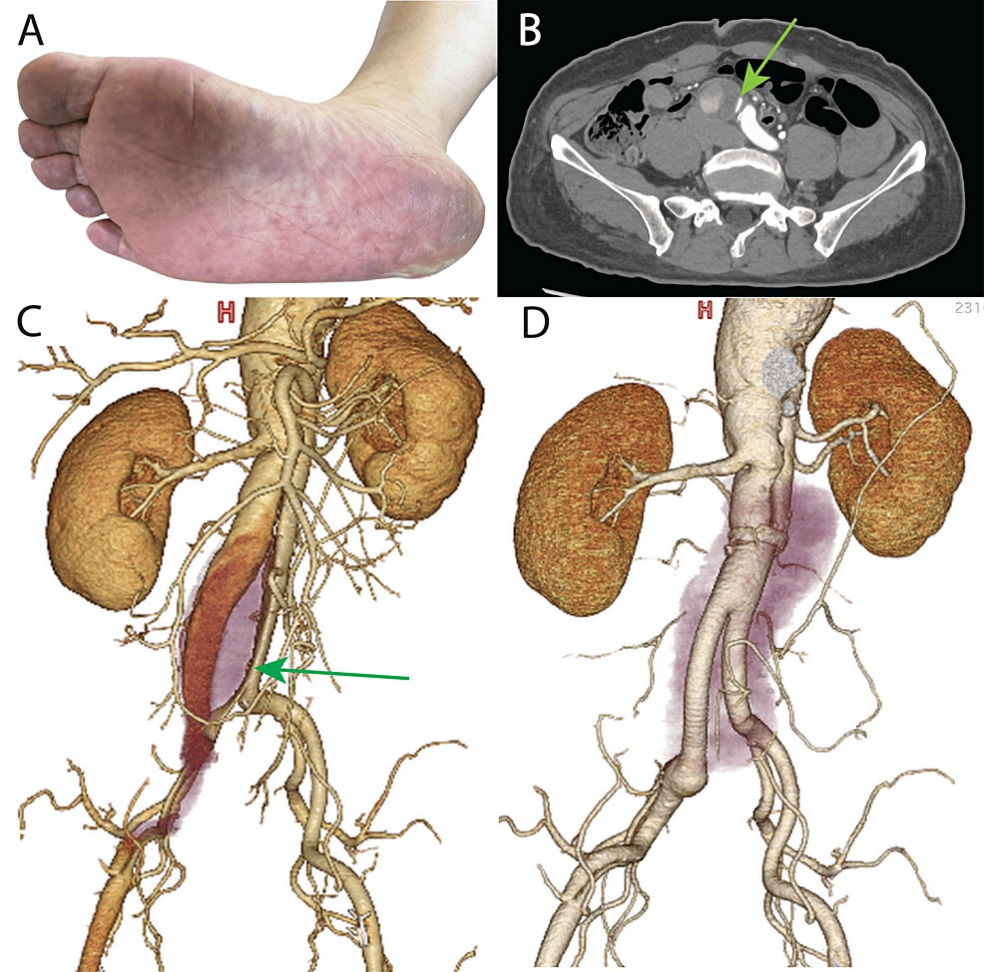
Preoperative findings (A) Discoloration of the sole of the foot was noted when symptoms of ischemia of the right lower extremity were present. The purple discoloration indicated ischemia. (B) Contrast-enhanced computed tomography at the appearance of symptoms of ischemia in the right lower extremity: the true lumen of the right common iliac artery was strongly compressed by the false lumen (arrow). (C) Contrast-enhanced computed tomography volume rendering before abdominal aortic artificial vascular replacement surgery: the abdominal aorta was dissected and extended to the right common iliac artery (arrow). The abdominal aorta itself had a diameter of 50 mm, which was an indication for surgery. (D) Contrast-enhanced computed tomography volume rendering after abdominal aortic artery replacement: the abdominal aorta was replaced with an artificial vessel.

Five years later, he developed ischemia of the right lower extremity (Figure 1A), which was considered to have been caused by ischemia of the lower extremity due to true lumen compression secondary to the enlargement of the false lumen of the right common iliac artery (Figure 1B). The abdominal aortic aneurysm was enlarged to 50 mm (Figure 1C), which was an indication for surgery.

**Figure 2:**
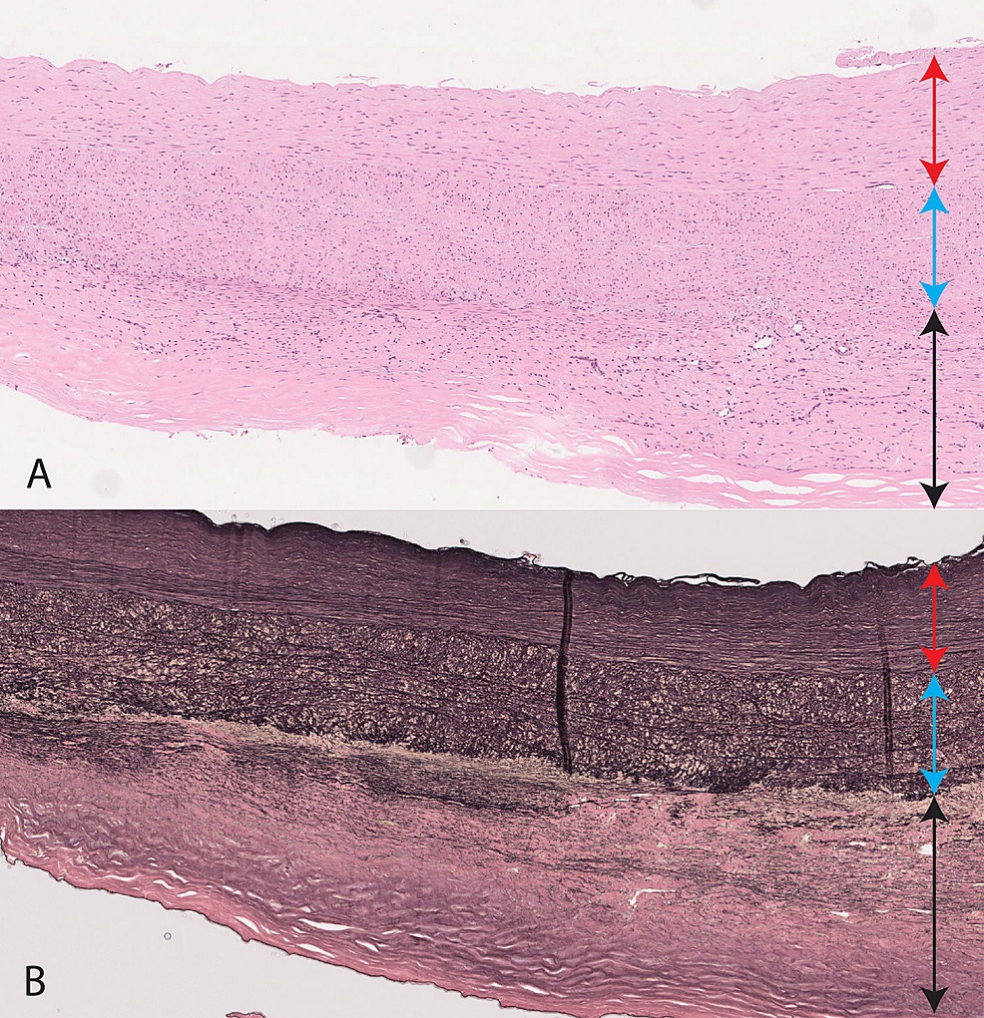
Pathological findings The red arrow is the intima, the blue arrow is the tunica media, and the black arrow is the neointima. (A) Hematoxylin and eosin stain. (B) Elastica van Gieson stain. Collagen fibers are seen in the neointima.

The operation was performed through a median abdominal incision with the patient under general anesthesia. No intra-abdominal adhesion was present. The retroperitoneal cavity was reached, and the abdominal aorta was secured below the renal artery. The peripheral portion of each common iliac artery was secured at its respective endpoint within the retroperitoneal space on both sides. Systemic heparin was administered, and the artery was clamped after confirming that the activated clotting time was >250 seconds. The artery was cut open, and the backflow from the lumbar artery was stopped. The central septum was resected to create a 1-cm-wide gap. The septum was then trimmed, and an artificial vessel (18- × 10-mm Hemashield Platinum; Maquet, Rastatt, Germany) was anastomosed. The left common iliac artery was anastomosed at the end, and blood flow was resumed. The right common iliac artery was found to be chronically dissected, and a 1-cm-wide section of the septal wall was resected and anastomosed end-to-side to resume blood flow. The septal wall and the outer wall of the vessel were submitted for pathological examination. Postoperative computed tomography showed no abnormalities at the anastomosis site, and the patient was discharged from the hospital on postoperative day 9 (Figure 1D). Pathological examination of the septal wall revealed thickened intima on both sides of the dissected tunica media (Figure 2A, 2B).

## Discussion

Chronic dissecting aortic aneurysm is a condition in which the false lumen enlarges in the chronic phase after acute aortic dissection [2]. Although conservative treatment that is focused on strict blood pressure control for acute type B aortic dissection without rupture or organ ischemia provides good results, many such cases are characterized by the enlargement of the aneurysm diameter in the chronic phase (aneurysmal enlargement of the false lumen) and may require surgical treatment to avoid rupture [3,4].

Research has shown that aortic dissection may play an important role in the weakening of the inner part of the aorta and that it is induced by malnutrition [5]. Although some reports have focused on the outer side of the vessel wall, no reports have addressed the chronic course of dissected septa [6]. In this case, we pathologically examined the septum of the abdominal aorta in a patient with chronic aortic dissection after five years of follow-up. Our findings indicated that, in general, immediately after aortic dissection, one side of the dissected septum was the tunica media and the other side was the intima; due to empirical changes, however, a new intima developed on the opposite side of the tunica media, in addition to the original intima. In such cases, these changes may be responsible for the increase in the thickness and firmness of the septum over time.

## Conclusions

A 51-year-old male underwent laparotomy for an abdominal aortic aneurysm associated with chronic aortic dissection. The septum between the true and false lumens was submitted to histopathological examination, which revealed bilateral intimal tissue on both sides of the tunica media. It was thought to be the original intima and neointima applied after dissection.
